# Identification and Characterisation of Infiltrating Immune Cells in Malignant Pleural Mesothelioma Using Spatial Transcriptomics

**DOI:** 10.3390/mps6020035

**Published:** 2023-03-28

**Authors:** Dmitrii Shek, Brian Gloss, Joey Lai, Li Ma, Hui E. Zhang, Matteo S. Carlino, Hema Mahajan, Adnan Nagrial, Bo Gao, Scott A. Read, Golo Ahlenstiel

**Affiliations:** 1 Blacktown Clinical School, Western Sydney University, Sydney, NSW 2148, Australia; 2 Westmead Institute for Medical Research, Sydney, NSW 2145, Australia; 3 Blacktown Mt Druitt Hospital, Sydney, NSW 2148, Australia; 4 Westmead Hospital, Sydney, NSW 2145, Australia; 5 Westmead Clinical School, University of Sydney, Sydney, NSW 2145, Australia; 6 Melanoma Institute Australia, Sydney, NSW 2065, Australia; 7 Institute of Clinical Pathology and Medical Research, NSW Health Pathology, Sydney, NSW 2145, Australia

**Keywords:** immune checkpoint inhibitors, mesothelioma, spatial analysis, research protocol

## Abstract

Increasing evidence strongly supports the key role of the tumour microenvironment in response to systemic therapy, particularly immune checkpoint inhibitors (ICIs). The tumour microenvironment is a complex tapestry of immune cells, some of which can suppress T-cell immunity to negatively impact ICI therapy. The immune component of the tumour microenvironment, although poorly understood, has the potential to reveal novel insights that can impact the efficacy and safety of ICI therapy. Successful identification and validation of these factors using cutting-edge spatial and single-cell technologies may enable the development of broad acting adjunct therapies as well as personalised cancer immunotherapies in the near future. In this paper we describe a protocol built upon Visium (10x Genomics) spatial transcriptomics to map and characterise the tumour-infiltrating immune microenvironment in malignant pleural mesothelioma. Using *ImSig* tumour-specific immune cell gene signatures and *BayesSpace* Bayesian statistical methodology, we were able to significantly improve immune cell identification and spatial resolution, respectively, improving our ability to analyse immune cell interactions within the tumour microenvironment.

## 1. Background

Malignant pleural mesothelioma (MPM) is a rare and aggressive tumour with less than 12 months median overall survival (OS) in advanced stages [1]. The incidence of MPM is 0.7 and 0.3 per 100,000 males and females, respectively, with a higher incidence in countries with greater exposure to asbestos such as Australia [2]. Most patients are diagnosed at an advanced stage, making them ineligible for curative surgery [1]. Consequently, systemic therapy has been a foundation for unresectable MPM [3] with the recently approved immune checkpoint inhibitors (ICIs) drastically increasing the OS by up to 18.4 months in MPM patients [4].

ICIs are monoclonal antibodies that stimulate the anti-tumour immune response by inhibiting checkpoint proteins such as cytotoxic T-lymphocyte-associated antigen 4 (*CTLA-4*), programmed cell death-1 (*PD-1*), its primary ligand *PD-L1* and other expressed on T cells, antigen-presenting cells (APCs) and cancer cells [5]. ICIs have already shifted treatment guidelines for various solid malignancies [6,7,8], although their tremendous efficacy has been challenged by a high incidence of immune-related adverse events (irAEs) which can affect up to 70% and 30% of patients treated with anti-CTLA-4 (Ipilimumab) [9] and anti-PD-1 agents (Pembrolizumab, Nivolumab), respectively [10]. The incidence of irAEs is dose-dependent with higher rates observed in patients treated with the combinatorial regimen of Ipilimumab (IPI) + Nivolumab (NIVO) [11]. Moreover, fatal irAEs can occur in 2% of patients and are refractory to steroids or other immunosuppressants [12].

Increasing evidence strongly supports the key role of the tumour microenvironment on response to systemic therapy. The tumour microenvironment is a complex tapestry of immune cells [13], some of which possess immunosuppressive roles by impairing T-cell infiltration and antigen presentation or by enhancing immunosuppressive metabolism [14]. This heterogeneity may impact ICI therapy outcomes, but remains poorly understood in this context, possessing the opportunity to reveal specific risk factors impacting ICI therapy. In this paper, we describe a protocol for using spatial transcriptomics to map the tumour-infiltrating immune microenvironment. We describe step-by-step guidance on how to combine Visium spatial technology with unique bioinformatic approaches to overcome the limitations of current Visium spatial gene expression and to enable the identification of immune cell populations at significantly enhanced resolution.

## 2. Experimental Design

### 2.1. Tissue Samples

Research sample collection and analysis was conducted as part of the NCT04631731 study (ICEMELT), which has been approved by the Western Sydney Local Health District (WSLHD) Human Research Ethics Committee (Ethics reference number: 2020/ETH02285). Malignant pleural mesothelioma patients were recruited at Blacktown and Westmead Public Hospitals in the WSLHD of Sydney, Australia. All patients provided written consent to participate in the study. A formalin-fixed paraffin-embedded (FFPE) tissue block was obtained from New South Wales Health Pathology (NSWHP), each containing tissue from the original tumour diagnostic biopsy prior to ICI therapy (*n* = 4).

### 2.2. Purchased Consumables

- Visium Spatial for FFPE Gene Expression kit, Human Transcriptome (10x Genomics, Pleasanton, CA, USA, 1000338);
- 10x Visium Accessory kit (10x Genomics, 1000194);
- Visium Tissue Section test slides (10x Genomics, 1000347);
- Dual Index kit TS Set A, 96 runs (10x Genomics, 1000251);
- RNeasy FFPE kit (Qiagen, Hilden, Germany, 73504);
- Deparaffinisation solution (Qiagen, 19093);
- Tween-20 (Sigma-Aldrich, St. Louis, MO, USA 9005-64-5);
- 10x phosphate-buffered saline pH 7.4 (ThermoFisher Scientific, Waltham, MA, USA, 70011609);
- 20x SSC buffer (ThermoFisher Scientific, 15557044);
- 8 M KOH (Merck, Darmstadt, Germany, 1310-58-3);
- 1 M Tris, pH 7.0, RNase free (ThermoFisher Scientific, AM9851);
- Low-Profile/triple facet microtome blades (Leica Biosystems, Wetzlar, Germany, 3802112);
- Kapa SYBR Fast qPCR Master Mix (2x), 1 mL (Sigma-Aldrich, KK4602);
- SPRIselect reagent, 450 mL (Beckman Coulter, Brea, CA, USA, B23319).

### 2.3. Equipment

- RM2125RT rotary microtome (Leica Biosystems);
- VS120 Slide Scanner (Olympus, Tokyo, Japan);
- Veriti 96-well thermocycler (Applied Biosystems, Waltham, MA, USA);
- 4200 TapeStation System (Agilent Technologies, Santa Clara, CA, USA).

### 2.4. Other Services

- Library quality control (QC) at AGRF (Australian Genome Research Facility, Melbourne, VIC, Australia);
- Sequencing at AGRF—Illumina Novaseq 6000 SP reagent kit v1.5, 50 bp PE.

## 3. Procedure

### 3.1. Overview of Workflow

#### 3.1.1. Case Selection and Tissue Preparation

For the purpose of this pilot study, *n* = 4 FFPE blocks from metastatic MPM patients were selected. All patients were treated with IPI + NIVO and coded as A1, B1, C1 and D1. The quality of the FFPE blocks was first assessed followed by pathological annotation. Biopsies of patients A1, B1 and C1 were obtained from the original tumour site, and in D1, the biopsy was obtained from a metastatic site in temporal lobe. One section of 5 µm thickness was cut from each block and stained with Hematoxylin & Eosin (H&E) for pathological review. The clinical pathologist examined the tissue to identify an appropriate high yield as the size of the tissue which can be mounted on a Visium slide is only 6.5 × 6.5 mm. The pathologist microscopically selected a 6.5 × 6.5 mm area of interest for this study, identifying the type of tissue (tumour, non-tumour, adipose tissue, immune cell infiltrates). For the purpose of our study, the area with a significant accumulation of infiltrating lymphocytes was chosen. Tumour core and periphery represent unique immune cell niches with altered cell densities that must be considered if performing comparative analysis among patient subgroups. We will elaborate on this concept in the discussion. The pathologist’s annotation was performed in one day and adjacent/tumour tissues within the examined area were characterised. Another 10 µm thick section from each block was cut for RNA extraction and quality control. Only samples with sufficient RNA quality, DV200 (percentage of RNA fragments of more than 200 nucleotides) > 50%, were used for this study.

#### 3.1.2. RNA Quality Check

RNA quality was measured from 10 µm sections extracted using the RNeasy kit from Qiagen as recommended by Visium guidelines. Briefly, FFPE tissue sections were deparaffinised, treated with Proteinase K (60 µg/mL) followed by heat treatment (56 °C for 15 min followed by 80 °C for 15 min), DNase (16 µL of DNase booster buffer and 10 µL of DNase I stock solution [2700 U/mL]), and transferred to Rneasy MinElute column. Following 2 wash steps, the quality of the eluted RNA was assessed using the Agilent TapeStation to obtain a DV200 score for each FFPE tissue. In our experience, tissue source and length of storage can significantly impact RNA quality for the purposes of spatial transcriptomics. Gastrointestinal tissue FFPE blocks, for example, are subject to significantly faster RNA degradation than MPM blocks stored for a similar time frame. All four MPM FFPE blocks obtained a sufficient DV200 score that was necessary to proceed.

#### 3.1.3. Visium Tissue Section Test Slide

Before placing sections on Visium slides, we first practiced mounting of the tissue sections onto 6.5 × 6.5 mm capture areas on regular Superforst Plus glass slides followed by staining with H&E to confirm adequate mounting and adhesion. We next followed the protocol provided by 10x Genomics using the Visium Tissue Section test slide to ensure adequate adhesion to the Visium slides. We observed very poor adhesion to these slides, with sections coming off the slides during H&E staining, even if processed delicately. We suspected that the absence of spatial barcode oligonucleotides impacted tissue adhesion, a complication that was confirmed by 10x Genomics representatives. We therefore decided to proceed with mounting our 4 sections onto the Visium Spatial Gene Expression slide. All sections adhered well to the Visium slides, suggesting that the bound oligonucleotides likely improve tissue adherence. These preliminary data impacted subsequent Visium guidelines, suggesting that users skip the tissue adhesion test for future experiments.

### 3.2. Histology and Imaging

**Figure 1:**
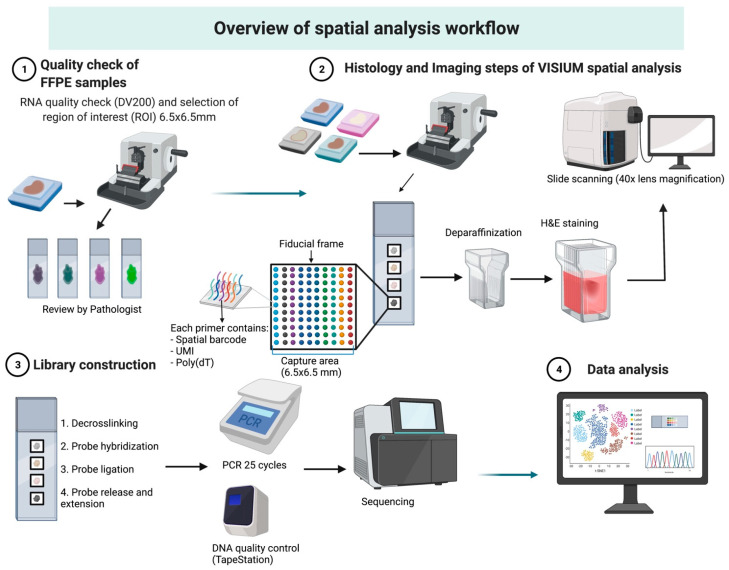
Overview of Visium spatial analysis workflow. Sections from FFPE tissues chosen for the spatial transcriptomic workflow are first quality checked, then freshly cut sections are adhered to the Visium slide. Following deparaffinisation and staining, they are imaged using a slide scanner followed by library construction, PCR amplification and sequencing. Spatial data are finally analysed using the Loupe Browser or other bioinformatic techniques as we describe herein. H&E—Hematoxylin & Eosin; UMI—unique molecular identifier.

The experimental protocol was scheduled to take 4 days (Table 1) beginning with a sectioning of FFPE blocks at a thickness of 5μm (Leica Biosystems Microtome). We strictly followed the protocol provided by 10x Genomics and no deviations were made. Each tissue section was placed within one of four 6.5 × 6.5 mm capture regions of the Visium slide. Each of the capture area contains an array of ~5000 spots (55 µm in diameter) which have oligonucleotide sequences required for the capture of probe-ligated mRNA, a spatial barcode, unique molecular identifier (UMI) and Illumina sequencing primer binding site (Figure 1). Sections were left overnight to dry and incubated the following day in a 60-degree fan-forced oven for 2 h and stained with H&E. The protocol for H&E staining was provided by 10x Genomics and was strictly followed. All reagents were newly purchased for the purpose of this analysis and were RNase-free. H&E-stained slides were coated with 85% glycerol and a coverslip, and imaged with the Olympus VS120 Slide Scanner at 40× lens magnification. After imaging, glycerol was washed off by immersing the Visium slide into the beaker containing 800 mL of Milli-Q water. The slide was immersed horizontally and held until the coverslip fully separated. Sections were dried at 37 °C and stored at 4 °C before decrosslinking.

### 3.3. Library Preparation and Sequencing

Samples were decrosslinked using the recommended TE buffer (pH 9.0) before human transcriptome probes covering 18,000 genes were ligated to target mRNA for capture and library construction according to the Visium Spatial Gene Expression for the FFPE protocol with no deviation. For PCR indexing, the optimal PCR cycle number determination was performed for each sample with the following cycles used in the final indexing PCR reaction: A1 (19 cycles), B1 (19 cycles), C1 (21 cycles) and D1 (18 cycles). DNA libraries were assessed using High Sensitivity D5000 ScreenTape on the TapeStation platform (Agilent Technologies), followed by sequencing using on a Novaseq SP flow cell with 50 bp paired end read length. The minimum sequencing depth required for each sample was calculated using the manual alignment tool in Loupe Browser to estimate the number of tissue-covered spots in each capture area and multiplying the spot number by the recommended 25,000 reads. On this basis, the minimum read pairs required for each sample were A1 (58.9 M reads), B1, (31.1 M reads), C1 (52.2 M reads) and D1 (90.4 M reads). Approximately 100 M paired reads were acquired for each sample and Fastq files were generated using the bcl2fastq 2.20.0.422 pipeline.

### 3.4. Bioinformatic Analysis

Gene expression counts were generated from fastq files using space ranger V1.3 with refdata-gex-GRCh38-2020-A and Visium Human Transcriptome Probe Set version 1.0 GRCh38-2020-A.csv annotations. Loupe files (generated from raw data using 10x Genomics Cloud Analysis software) were analysed using loupe browser and count matrices were imported into R for analysis with *Seurat* [15]. Clustering and spatial enhancement was performed using *BayesSpace* according to the author’s instruction [16]. Briefly, optimised clustering resolution was determined using the qtune function (between 5–7 clusters per section), and the optimised enhancement parameter jitter_scale was determined using the mcmcChain function on low replicate (*n* = 300) spatial-enhanced data; for our sections, the optimum scale was 0.35. Spatial enhancement was performed using these optimised parameters for 10,000 repetitions.

To define the tumour-infiltrating immune cells within the spatial framework, gene expression signature lists were used from the R package *ImSig* [17] for immune cells (B cells, T cells, NK cells, neutrophils, macrophages and monocytes). To define MPM tumour tissue, a gene list containing 51 overexpressed genes identified in MPM was used (e.g., MSLN, ALDOA, CDC2, NMU, PCNA, PDGFRB) from a systematic review of 10 independent transcriptome studies [18]. Enhanced feature expression was generated for all nominated genes (*n* = 401) using *BayesSpace,* and for each feature, a score was generated using the sum of scaled expression normalised to the size of the gene list and overall gene expression for that feature. Cell types were then assigned to features by scores greater than the background and verified by manual image analysis. A manual cut-off of 250 was used on the scores enhanced by the expression scores that were scaled, using the sum of the signature gene scaled expression values divided by the number of genes in each individual signature [19]. Neighbour counting was carried out using a custom script in R. Briefly, for each feature, all features sharing at least one vertex (up to 12 per feature) were evaluated for cell type assignments and tallied for each. In addition, Loupe browser version 6.0.0 (10x Genomics) was used for analysis of the spatial data. ConsensusPath-DB online software was used to functionally annotate the set of significant (*p* < 0.05) DEGs. Graphical summaries of immune cell compositions were completed using Prism version 9 (GraphPad Software, LLC). Statistics were not performed on this data due to insufficient power (*n* = 4 sections).

## 4. Results

**Figure 2:**
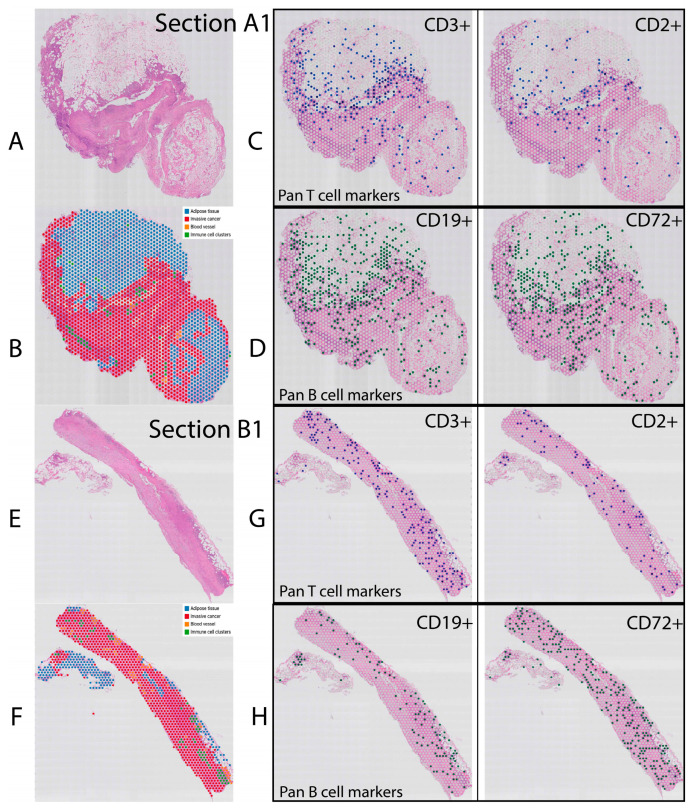
H&E and spatial images of analysed tissues using *Loupe browser*. H&E images of tissues A1 and B1 scanned with Olympus slide scanner (**A**,**E**) and pathologist’s annotation of tissues A1 and B1 in Loupe browser (**B**,**F**). To identify immune cells within the tissue sections, pan-T-cell (**C**,**G**) and pan-B-cell (**D**,**H**) markers were queried in Loupe browser. Discordance of spots assigned to T and B cells using pan-cell markers represent a major limitation of cells identification in Loupe browser.

Tissue sections (6.5 mm × 6.5 mm) from each block were processed to quantify spatial gene expression (VISIUM, 10x Genomics) offering 9041 barcoded arrays across four samples with an average of 3125 genes detected per spot. The summary reports from Space Ranger (10x Genomics) are presented in Supplementary Materials (Figure S1). Figure 2A,B,E,F demonstrate H&E staining as well as the pathologist’s annotation of tissue sections A1 and B1, demonstrating the heterogeneity of tissue as well as immune cell aggregation in tumour tissue. The goal of this study was to identify and transcriptionally characterise immune cells within the tumour landscape. Unfortunately, this was not immediately feasible for two reasons: low spatial resolution and depth of sequencing. The 55 µm spot diameter present on the Visium slides contained transcriptional data from multiple cells, resulting in mixed-cell transcriptomes. This issue was particularly problematic when identifying immune cell clusters, representing densely packed areas of multiple immune cell populations. This was evident in tissues A1 and B1, where immune cells aggregated at the tumour periphery. Secondly, the depth of sequencing was not sufficient to identify immune cells based on the expression of a single gene (e.g., CD3+ T cells, CD19+ B cells or CD56+ NK cells). Moreover, different immune cell-specific transcripts would generate significantly different spatial maps of immune cell location (Figure 2C,D,G,H). For example, pan-T-cell markers CD3 and CD2 generated significantly different T-cell maps, with CD3 generating denser T-cell distribution containing only partial overlap with CD2.

**Figure 3:**
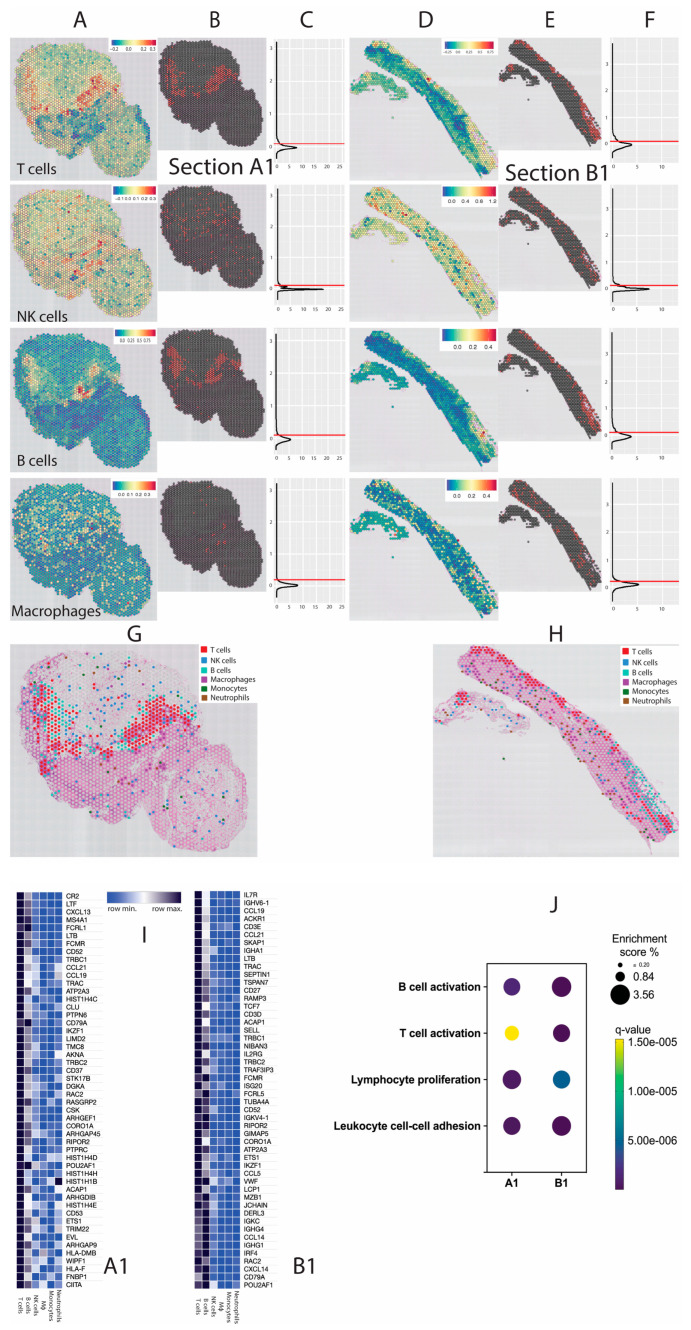
Identification of tumour-infiltrating immune cells using the *ImSig* algorithm in tissues A1 and B1. (**A**,**D**,**G**,**H**) Loupe images demonstrating infiltrating immune cells identified using the *ImSig* algorithm in tissue A1 and B1 respectively. (**B**,**E**) R images representing the selection of custom cut-off values for each type of immune cell(s). (**C**,**F**) Histograms represent the cut-off values assigned to each subtype of immune cells using *Seurat* function. *AddModuleScore* was used to score features for signatures and made a manual cut-off for each signature to exclude the negative population (a la flow). (**I**) Heatmaps representing the top 50 significantly upregulated genes in T-cell spots compared to non-T-cell spots from sections A1 and B1. The relative expression of these genes in other *Imsig*-based immune cell spots indicates that there is significant overlap in B-cell and T-cell identification due to non-single cell resolution of the Visium slide. (**J**) Pathways enriched by the top 50 differentially expressed genes in T cells of tissues A1 and B1. Again, we can observe an overlap with B-cell related pathways emphasising the limitation of the Visium spatial method.

To overcome the limitation of immune cell identification, we used *ImSig* tumour-specific immune cell gene signatures [17] that enabled us to confidently identify tumour infiltrating immune cell populations (T cells, B cells, monocytes, macrophages, neutrophils, natural killer [NK] cells) (Figure 3). In addition to immune cells, *ImSig* enabled the identification of other cell signatures including interferon, proliferation and translation that are intricately linked to oncogenic signalling and anti-cancer responses [17]. Applied to the spatially resolved data, *ImSig*, immune cell transcriptomes and locations could be examined and compared within sections and between sections based on a variety of clinical parameters including treatment response and/or ICI toxicity. Due to limitations of the Visium slide, some spots were assigned to multiple immune cell types. We manually labelled each of the spots sequentially, beginning with T cells, followed by B cells, NK cells, macrophages, monocytes and neutrophils (Figure 3G,H).

**Figure 4:**
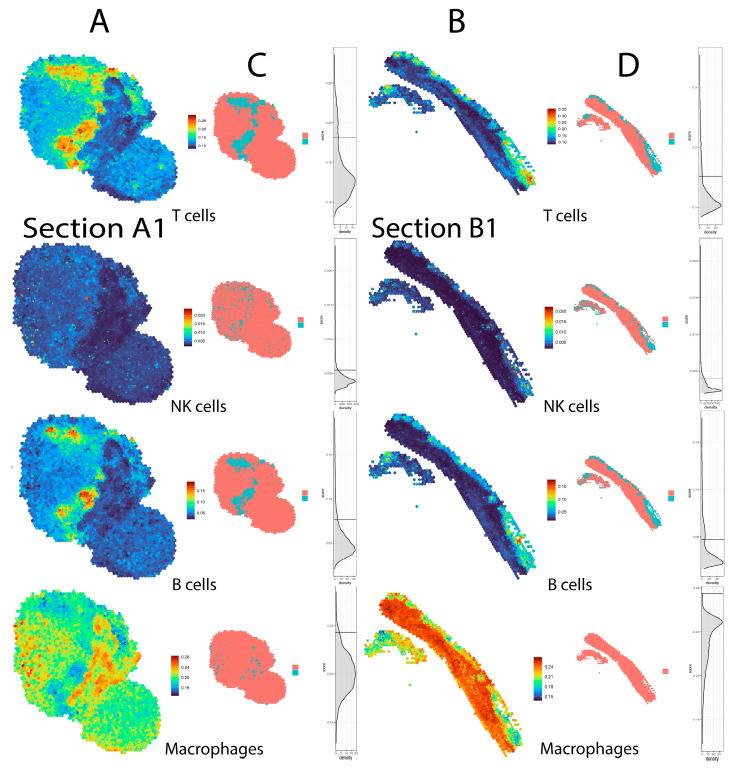
Increased resolution of spatial images with *BayesSpace* method. (**A**,**B**) Enhanced resolution allowed us to more precisely locate and quantify tumour-infiltrating immune cells, particularly T cells, B cells, NK cells and macrophages using the *ImSig* algorithm. (**C**,**D**) Scales and histograms represent the cut-off values assigned to elucidate the signature scores for each section and/or cell type using an outlier analysis (hampel filter) [20].

To address the spatial limitation of the Visium method, we used the *BayesSpace* package that utilises the Bayesian statistical method to subdivide each spatial spot and obtaining sub-spot resolution. *BayesSpace* uses transcriptional data from neighbouring spots to increase the resolution of each 55 µm spot 6-fold. As immune cell sizes are in the range of ~5–20 µm and parenchymal cells are even larger, this method increased the resolution of our sections to a nearly single-cell level. Enhancement of data allowed a more precise identification of immune cell clusters within the tumour (Figure 4). BayesSpace resolved the tissue structure that is not detectable at original resolution and applicable both to infiltrating immune cells and malignant cells (Figure S2), thus overcoming a major limitation of the current Visium spatial technology.

**Figure 5:**
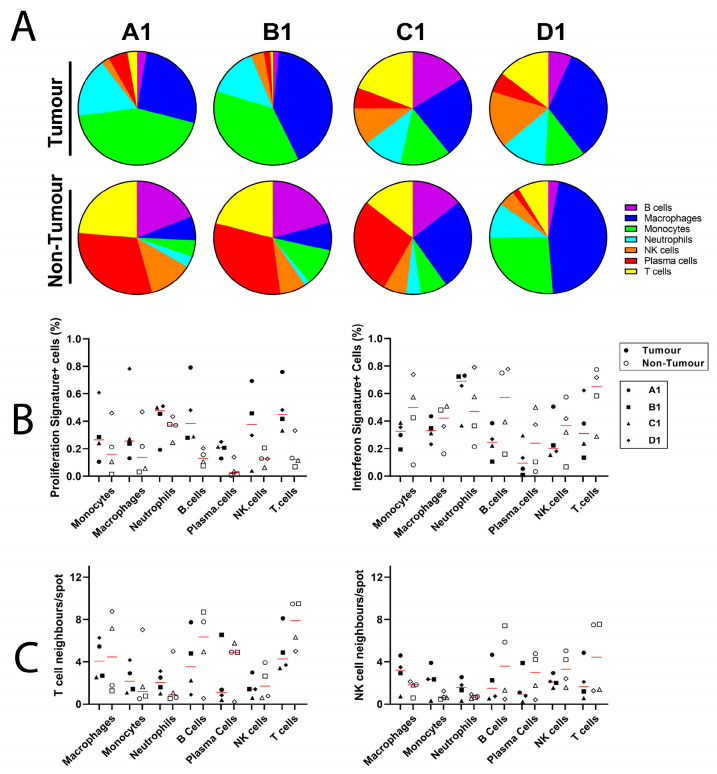
Analysis of *BayesSpace*-enhanced immune cell composition. (**A**) *Imsig*-characterised immune cell composition of *BayesSpace* enhanced tissue sections A1-D1 showing differences between tumour and adjacent non-tumour tissue. (**B**) Proportion of *ImSig*-characterised immune cell spots sharing proliferation and interferon signatures in tumour and adjacent non-tumour tissue. (**C**) Analysis of T- and NK-cell neighbouring spots among different immune cell populations in tumour and adjacent non-tumour tissue.

We next sought to analyse immune cell makeup, phenotype and interactions in normal and tumour tissue using *BayesSpace*-enhanced images, with which we have generated coordinate-based information for all *ImSig*-based annotation. Figure 5A demonstrates the immune cell makeup of tumour and non-tumour tissue based on the *ImSig* identification of immune cells. Both A1 and B1 sections demonstrate drastically different immune cell compositions, dominated by myeloid cells in the tumour and lymphocytes in adjacent tissue. Conversely, the immune cell composition within C1 and D1 are more similar within tumour and adjacent tissue. Tumour and adjacent immune cell populations were next characterised based on whether they shared proliferation or interferon signatures defined using *ImSig* (Figure 5B). The proportion of immune cell spots sharing the proliferation signature was largely increased in the tumour tissue, whereas the interferon signature was generally enhanced in the adjacent non-tumour tissue immune cells. Lastly, immune cell interactions were quantified by calculating the average number of NK and T-cell neighbouring spots held by individual immune cell populations, with a maximum of 13 neighbours (including co-localisation) following *BayesSpace* enhancement (Figure 5C). Both T- and NK-cell spots generally demonstrated more interactions with myeloid cell populations (monocytes, macrophages and neutrophils) in tumour tissue and fewer interactions with other lymphocyte populations (B, T and NK cells). These data provide crucial information regarding immune cell interactions with potential clinical implications; however, the limited sample size and significant variability among sections used in this study have limited any further statistical considerations.

## 5. Discussion

Spatial transcriptomics has recently been declared the Method of Year by the *Journal Nature Methods* [21]. Spatially resolved transcriptomics enables the characterisation of transcriptional patterns within sections of tissue while preserving the original tissue architecture [22]. It overcomes the limitations of bulk and single-cell sequencing methods which can provide the transcriptomic data without tissue alignment. A large study (516 MPM samples) conducted by Aday et al., for example, established that T_H2_ and cytotoxic T cells are abundant in tumours of patients with greater overall survival [23]. Their study was nonetheless unable to identify cellular identity and phenotype, its relation to neighbouring cells and non-cellular structures which provides crucial information regarding tumour composition and the role of infiltrating lymphocytes in treatment response [24]. There are a few commercialized spatial techniques that can be used on both FFPE and fresh–frozen tissues [22]. Herein, we describe a methodology of using a Visium spatial transcriptomics method (10x Genomics) on FFPE tissue samples collected from a real-world cohort of cancer (MPM) patients. In this pilot study we were able to successfully implement a Visium-based framework to identify and characterise tumour-infiltrating immune cells in FFPE tissues using various packages in R.

While Visium spatial transcriptomics is a powerful technology, there are limitations of the current slides that must be noted. Most importantly, the diameter of each spatial spot under the tissue section is 55 µm, which will likely capture transcripts from multiple cells, thus diluting important information regarding cellular identity and phenotype. BayesSpace was able to increase resolution 6-fold; however the spatial enhancement comes at the cost of greatly increasing the amount of data produced and a greater reliance on orthogonal validation. Most importantly, the resolved images could not be exported to the Loupe browser and aligned with gene expression data. Consequently, differential gene expression could not be performed. Nonetheless, the next generation of spatial kits offered by 10x Genomics provide better resolution (much smaller spot size) with a larger tissue capture area [25]. This will surely simplify downstream analysis; however, cell identification tools such as *ImSig* will likely still be necessary to identify cell populations.

The second major limitation of spatial transcriptomics technology is the absence of open-source and well-documented software for performing data analysis. A creation of a unique bioinformatic tool for data collection and analysis will markedly simplify the workflow and allow researchers to focus more on understanding the biological problem rather than spending time learning new syntax or bringing in seasoned bioinformaticians to implement a new algorithm/code using *R*-based frameworks. In the near future, more advanced tools and techniques will likely appear to provide more in-depth analysis of spatial data, thus dramatically changing the field of clinical and biomedical research. Moreover, spatially resolved transcriptomics have a potential to be introduced into clinical diagnostic guidelines as a high-throughput tool for guiding physicians’ decision making.

Tissue acquisition and selection for spatial profiling represents an additional hurdle that can significantly impair analyses as performed herein. Extensive planning and communication with medical staff is necessary to obtain tumour tissue that is well characterised histologically with regard to location within the tumour (central versus peripheral). The retrospective acquisition of FFPE biopsy tissue represents a significant limitation of this study. Biopsy tissue was obtained from different locations within the tumour, and hence possesses different immune cell profiles. Sections A1 and B1 are clearly isolated from a peripheral tumour location, as demonstrated by adipose tissue surrounding the tumour. Indeed, these sections demonstrate significantly different immune cell profiles and interactions as compared to sections C1 and D1 that are more homogenous with regard to tissue composition. Future studies sampling homogenous tumour locations will better inform how clinical parameters (e.g., treatment response, toxicity) are affected by the tumour microenvironment.

In summary, the preservation of tissue architecture and alignment with gene expression data allowed us to determine number and location of tumour-infiltrating immune cells and the upregulation of genes potentially responsible for better/lower therapeutic outcomes in examined patients. Further exploration of cell–cell interactions by implementing spatial and single-cell methods is necessary, and in the near future, it may identify the unique cellular markers correlated with immunotherapy outcomes in patients with rare cancers.

## Figures and Tables

**Table 1:** A step-by-step plan of the Visium workflow. D—day; FFPE—formalin-fixed paraffin-embedded; H&E—hematoxylin and eosin.

Step	Sub-Steps	Location	Estimated Timing
Case selection and quality control	Selection of FFPE blocks Sectioning and H&E staining for pathological review Assess RNA quality	Pathology Lab Histology Lab Biobank	D0 9:00–10:30 D0 10:30–2:00 D0 3:00–6:00
FFPE Tissue Sectioning and Placement on Visium Slide	Scale and section Place section on Visium slide	Histology Lab Histology Lab	D1 9:00–9:30 D1 10:30–1:30
Deparaffinisation, H&E Staining, Imaging, Decrosslinking	Prepare buffers Deparaffinisation H&E staining Tissue imaging Coverslip removal Decrosslinking	Histology Lab Histology and Genomics Labs Histology Lab Cell Imaging Lab Histology and Genomics Labs Genomics Lab	D2 9:00–9:30 D2 9:00–12:00 D2 12:00–12:15 D2 12:30–1:00 D2 1:00–1:25 D2 2:00–3:15
Probe hybridisation	Probe hybridisation Probe ligation Probe release and extension	Genomics Lab Genomics Lab Genomics Lab	D2 3:15–3:45 D3 9:00–12:00 D3 1:00–3:00
FFPE Library construction	qPCR cycle number determination Sample index PCR Clean-up Library QC	Genomics Lab Genomics Lab Genomics Lab Genomics Lab	D4 9:00–10:00 D4 10:00–11:00 D4 11:00–11:30 D4 11:30–12:00

## Data Availability

The data are available upon a request to a corresponding author.

## Supplementary Materials

The following supporting information can be downloaded at: https://www.mdpi.com/article/10.3390/mps6020035/s1, Figure S1: Technical Summary reports of spatially resolved transcriptomics datasets; Figure S2: Identification of malignant cells in examined tissues.
