# Supplementary material for: Identification and Characterisation of Infiltrating Immune Cells in Malignant Pleural Mesothelioma Using Spatial Transcriptomics
Source: Methods Protoc. 2023 Mar 28;6(2):35. doi: 10.3390/mps6020035 (PMC10146955; doi:10.3390/mps6020035)
Supplement: Supplementary file 1 [file mps-06-00035-s001.zip › mps-2250518-supplementary.pdf]

methods and protocols

MDPI

Supplementary materials

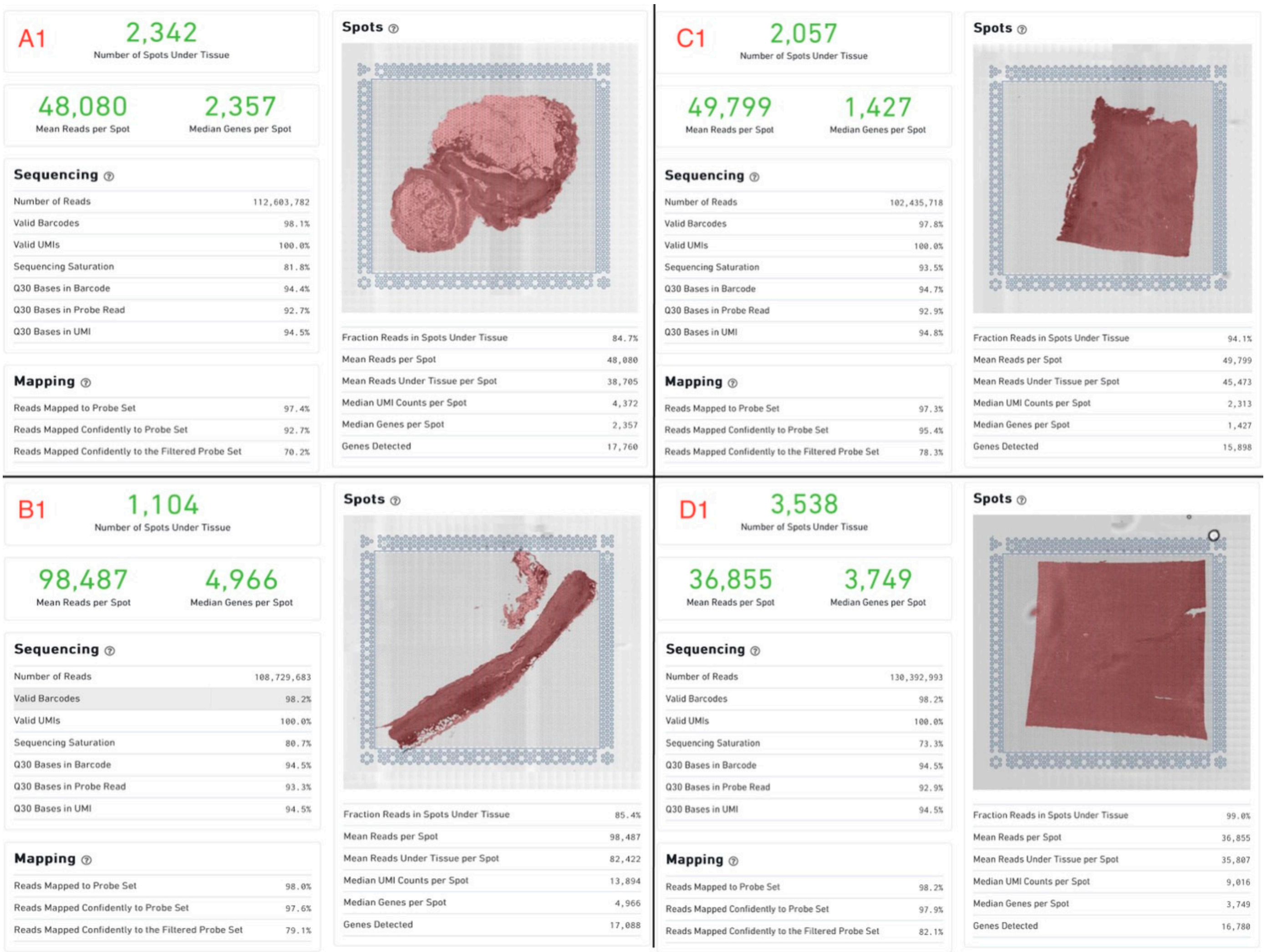

Figure S1. Technical Summary reports of spatially resolved transcriptomics datasets. Reports were obtained from Space Ranger software (10X Genomics) demonstrating sequencing efficiency and coverage. We detected a median of 6,694 Unique Molecular Identifier (UMI) counts per spot across all studied samples with a mean of 50,601 reads under tissue per spot.

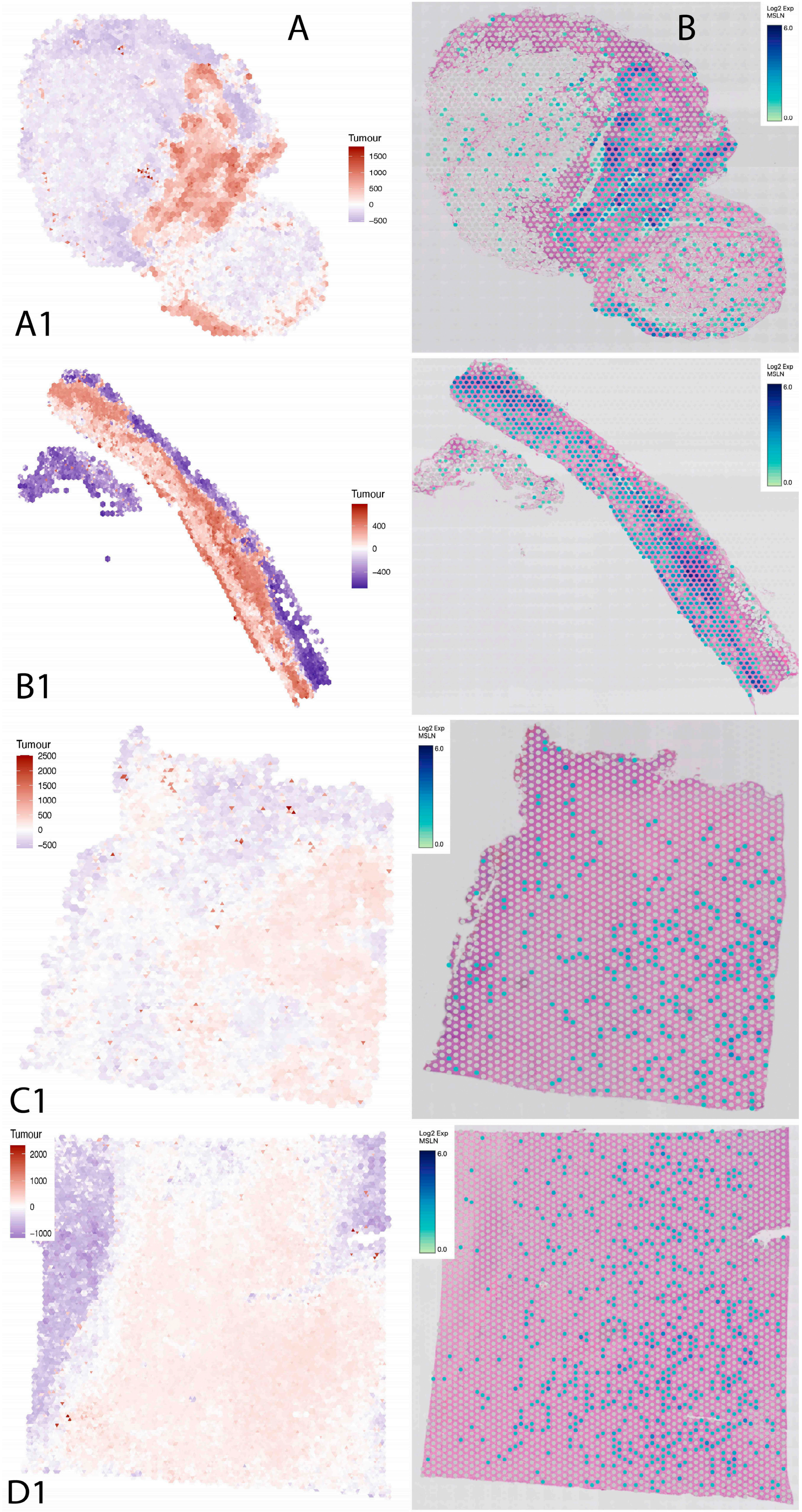

Figure S2. Identification of malignant cells in examined tissues. A – Tumour cells identified witrh BayesSpace (a set of genes was used to precisley locate malignant cells). B – Tumour cells identified with Loupe browser using *MSLN* (mesothelin) gene as a specific MPM gene.
